# Reduced RKIP enhances nasopharyngeal carcinoma radioresistance by increasing ERK and AKT activity

**DOI:** 10.18632/oncotarget.7201

**Published:** 2016-02-05

**Authors:** Li Yuan, Hong-Mei Yi, Hong Yi, Jia-Quan Qu, Jin-Feng Zhu, Li-Na Li, Ta Xiao, Zhen Zheng, Shan-Shan Lu, Zhi-Qiang Xiao

**Affiliations:** ^1^ Research Center of Carcinogenesis and Targeted Therapy, Xiangya Hospital, Central South University, Changsha, Hunan 410008, China; ^2^ The Higher Educational Key Laboratory for Cancer Proteomics and Translational Medicine of Hunan Province, Xiangya Hospital, Central South University, Changsha, Hunan 410008, China

**Keywords:** nasopharyngeal carcinoma, radioresistance, RKIP, ERK−1/2, AKT

## Abstract

Raf kinase inhibitory protein (RKIP) functions as a chemo-immunotherapeutic sensitizer of cancers, but regulation of RKIP on tumor radiosensitivity remains largely unexplored. In this study, we investigate the role and mechanism of RKIP in nasopharyngeal carcinoma (NPC) radioresistance. The results showed that RKIP was frequently downregulated in the radioresistant NPC tissues compared with radiosensitive NPC tissues, and its reduction correlated with NPC radioresistance and poor patient survival, and was an independent prognostic factor. *In vitro* radioresponse assay showed that RKIP overexpression decreased while RKIP knockdown increased NPC cell radioresistance. In the NPC xenografts, RKIP overexpression decreased while RKIP knockdown increased tumor radioresistance. Mechanistically, RKIP reduction promoted NPC cell radioresistance by increasing ERK and AKT activity, and AKT may be a downstream transducer of ERK signaling. Moreover, the levels of phospho-ERK−1/2 and phospho-AKT were increased in the radioresistant NPC tissues compared with radiosensitive ones, and negatively associated with RKIP expression, indicating that RKIP-regulated NPC radioresponse is mediated by ERK and AKT signaling in the clinical samples. Our data demonstrate that RKIP is a critical determinant of NPC radioresponse, and its reduction enhances NPC radioresistance through increasing ERK and AKT signaling activity, highlighting the therapeutic potential of RKIP-ERK-AKT signaling axis in NPC radiosensitization.

## INTRODUCTION

Nasopharyngeal carcinoma (NPC) is prominent in a number of Southeast Asian regions, and poses a very serious health problem in these areas [1]. Radiotherapy is a preferred and widely used modality for treatment of NPC, and most NPC patients can be cured if the disease is diagnosed and treated at an early stage. However, about 30% of NPC patients develop local recurrence and distant metastasis after radiotherapy duo to radioresistance, which is a major cause of treatment failure in many cases [2, 3].

Raf kinase inhibitory protein (RKIP) is a small evolutionary conserved protein that was first identified as a physiological inhibitor of Raf kinase, antagonizing Raf-1/MEK/ERK pathway [4]. Actually, RKIP has been implicated in multiple intracellular signaling pathways [5–8] that regulate various biological processes [9]. RKIP is well known for its metastasis suppression function [10], being its loss or reduced expression associated with metastasis and prognosis in many types of cancers [11–15]. Studies have showed that reduced RKIP correlates with the chemoresistance of cervical cancer [16], lung adenocarcinoma [17], gastric cancer [18] and prostate and breast cancer [19], and upregulation of RKIP expression increases the sensitivity of cancer cells to chemotherapy [16–20]. Moreover, RKIP expression level also correlates with immunotherapeutic response, and induction of RKIP expression results in the sensitization of resistant cancer cells to immunotherapy [20–23]. However, the role of RKIP in tumor radioresistance was rarely reported [24, 25]. In our previous comparative proteomic study, RKIP was identified as one of reduced proteins in the NPC tissues as compared to normal nasopharyngeal mucosal tissues [26]. Recently, we demonstrated that RKIP is a potential metastatic suppressor of NPC [27]. However, the role and signaling mechanism of RKIP in NPC radioresistance remain unclear.

Ionizing radiation not only causes DNA double-strand breaks (DSBs) of cancer cells, inducing cell apoptosis, but also generates reactive oxygen species (ROS), leading to activation of multiple prosurvival and anti-apoptotic signaling pathways, such as MEK-ERK and PI3K-AKT pathways, which play a role in the cell survival from toxic stresses and the regulation of cell growth [28, 29]. As RKIP is an endogenous inhibitor of Raf-MEK-ERK pathway [4], RKIP downregulation will result in imbalance between activation and inhibition of MEK-ERK in the irradiated cells. The previous study also suggested that ROS stimulates cell proliferation in the palmitic acid-treated hepatocytes by activating ERK-AKT signaling pathway, in which AKT is a downstream transducer of ERK signaling [30]. Based on these reasons, we hypothesize that RKIP reduction increases NPC radioresistance by decreasing its inhibition on ERK and AKT signaling.

In this study, we first detected RKIP expression in the radiosensitive and radioresistant NPC tissues, and evaluated its correlation with the radioresistance and clinical outcomes of NPC patients. Secondly, with a combination of loss-of-function and gain-of-function approaches, we analyzed the function of RKIP in NPC radioresponse, and assessed if reduced RKIP confers NPC cells radioresistance *in vitro* and *in vivo*. Finally, we determined whether RKIP-regulated NPC cell radioresponse is mediated through ERK and AKT signaling. Here, we report that RKIP reduction is associated with NPC radioresistance and poor patient prognosis, and reduced RKIP promotes NPC radioresistance by increasing ERK and AKT signaling activity.

## RESULTS

### RKIP reduction is associated with NPC radioresistance and poor patient prognosis

**Figure 1:**
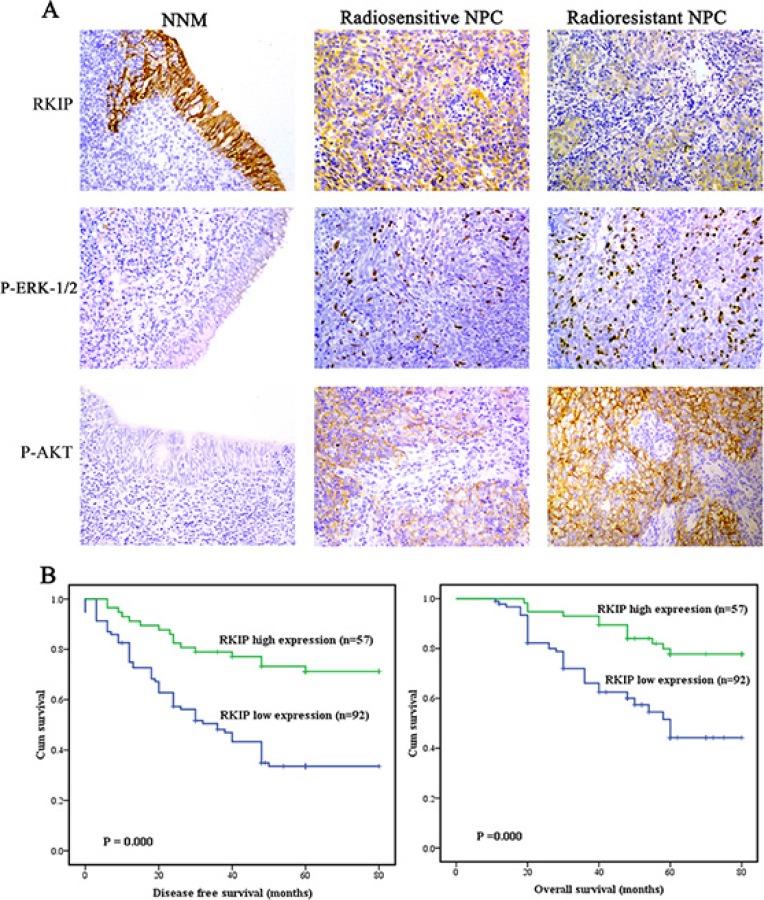
RKIP reduction is correlated with NPC radioresistance and poor patient survival (**A**) a representative result of RKIP, phospho-ERK−1/2 and phospho-AKT immunohistochemical staining in normal nasopharyngeal mucosa (NNM), and radiosensitive and radioresistant NPC tissues. Original magnification, ×200. (**B**) Kaplan-Meier survival analysis for NPC patients according to the expression levels of RKIP. NPC patients with low RKIP expression have a significantly worse disease-free survival (left) and overall survival (right) than those with high RKIP expression.

Immunohistochemistry was performed in a cohort of NPC tissues including 74 radioresistant and 75 radiosensitive NPCs and 30 normal nasopharyngeal mucosal (NNM) tissues. We observed that RKIP expression was dramatically reduced in the NPCs relative to NNM, and in the radioresistant NPCs relative to radiosensitive NPCs (Figure 1A, Table 1). The relationships between RKIP expression and clinicopathologic variables are summarized in Table 2. As shown in this table, the expression levels of RKIP were negatively correlated with NPC radioresistance (*r* = −0.489, *P* < 0.001), and tumors with low RKIP expression were frequently radioresistant. Moreover, the expression levels of RKIP were correlated with lymph node metastasis and TNM stage.

**Table 1:** The expression levels of RKIP, phospho-AKT and pospho-ERK−1/2 in the NNM and NPCs with different radioresponse

Protein	NNM (*n*, %)	Radiosensitive NPC (*n*, %)	Radioresistant NPC (*n*, %)
**RKIP**			
Low (0–3)	5 (16.67)	28 (37.84)	64 (85.33)
High (4–6)	25 (83.33)	46 (62.16)	11 (14.67)
**Phospho-AKT**			
Low (0–3)	30 (100.00)	44 (59.46)	23 (30.67)
High (4–6)	0 (0.00)	30 (40.54)	52 (69.33)
**Phospho-ERK**−**1/2**			
Low (0–3)	29 (96.67)	42 (56.76)	26 (34.67)
High (4–6)	1 (3.33)	32 (43.24)	49 (65.33)

NNM, normal nasopharyngeal mucosa; NPC, nasopharyngeal carcinoma.

*P* < 0.01 by χ^2^ test, NNM *vs*. NPC; Radiosensitive NPC *vs*. Radioresistant NPC.

**Table 2:** Correlation between RKIP expression and clinicopathological characteristics in nasopharyngeal carcinoma (*n* = 149, χ^2^ test)

Variables	N	RKIP		
		low	high	*P*
**Gender**				
Male	110	67	43	
Female	39	25	14	0.724
**Age (y)**				
< 47	68	42	26	
≥ 47	81	50	31	0.996
**Primary tumor (T) stage**				
T1–2	74	43	31	
T3–4	75	49	26	0.364
**Lymph node (N) metastasis**				
N0	63	33	30	
N1–3	86	59	27	0.044
**Clinical TNM stage**				
I–II	41	17	24	
III–IV	108	75	33	0.002
**Radiation response**				
Sensitivity	74	28	46	
Resistance	75	64	11	0.000

Since radioresistance is a major cause that leads to the poor outcomes of NPC patients, we analyzed the ability of RKIP to predict disease free survival (DFS) and overall survival (OS) of the patients. Survival analysis revealed that low RKIP level in NPCs correlated with the markedly reduced DFS and OS of the patients (Figure 1B). A univariate Cox proportional hazards regression analysis showed that RKIP expression level and clinical TNM stage significantly affected the DFS and OS of NPC patients (Table 3). A multivariate Cox proportional hazards regression analysis confirmed that low RKIP expression was an independent predictor for the reduced DFS and OS of NPC patients (Table 3). These results indicate the importance of RKIP expression level in the clinical NPC radioresistance.

**Table 3:** Univariate and multivariate analyses of prognostic factors for overall and disease-free survival using Cox proportional hazards regression model (*N* = 149)

Variable	Disease free survival (DFS)				Overall survival (OS)			
	Univariate analysis		Multivariate analysis		Univariate analysis		Multivariate analysis	
	*P*	HR (95% CI)	*P*	HR (95% CI)	*P*	HR (95% CI)	*P*	HR (95% CI)
**Gender**								
Male vs. Female	0.125	0.642 (0.365–1.131)	0.285	0.731 (0.411–1.298)	0.607	0.849 (0.454–1.587)	0.969	0.987 (0.518–1.881)
**Age(y)**								
< 47 vs. ≥ 47	0.947	1.016 (0.644–1.601)	0.876	0.963 (0.601–1.544)	0.620	1.148 (0.665–1.983)	0.900	1.037 (0.587–1.832)
**Primary tumor(T) stage**								
T1–2 vs.T3–4	0.215	1.336 (0.845–2.111)	0.084	0.638 (0.383–1.063)	0.542	1.183 (0.690–2.029)	0.056	0.569 (0.319–1.015)
**lymph node(N) metastasis**								
N0 vs.N1–3	0.006	2.016 (1.225–3.319)	0.290	0.706 (0.371–1.344)	0.018	2.062 (1.131–3.756)	0.105	0.530 (0.246–1.143)
**Clinica TNM stage**								
I–II vs. III–IV	0.000	4.320 (2.072–9.006)	0.027	3.023 (1.131–8.078)	0.000	6.335 (2.281–17.594)	0.023	4.449 (1.230–16.091)
RKIP expression level								
Low vs. High	0.000	0.320 (0.184–0.558)	0.002	0.414 (0.235–0.728)	0.000	0.311 (0.165–0.589)	0.004	0.377 (0.196–0.727)

### RKIP reduction increases NPC cell radioresistance *in vitro*

**Figure 2:**
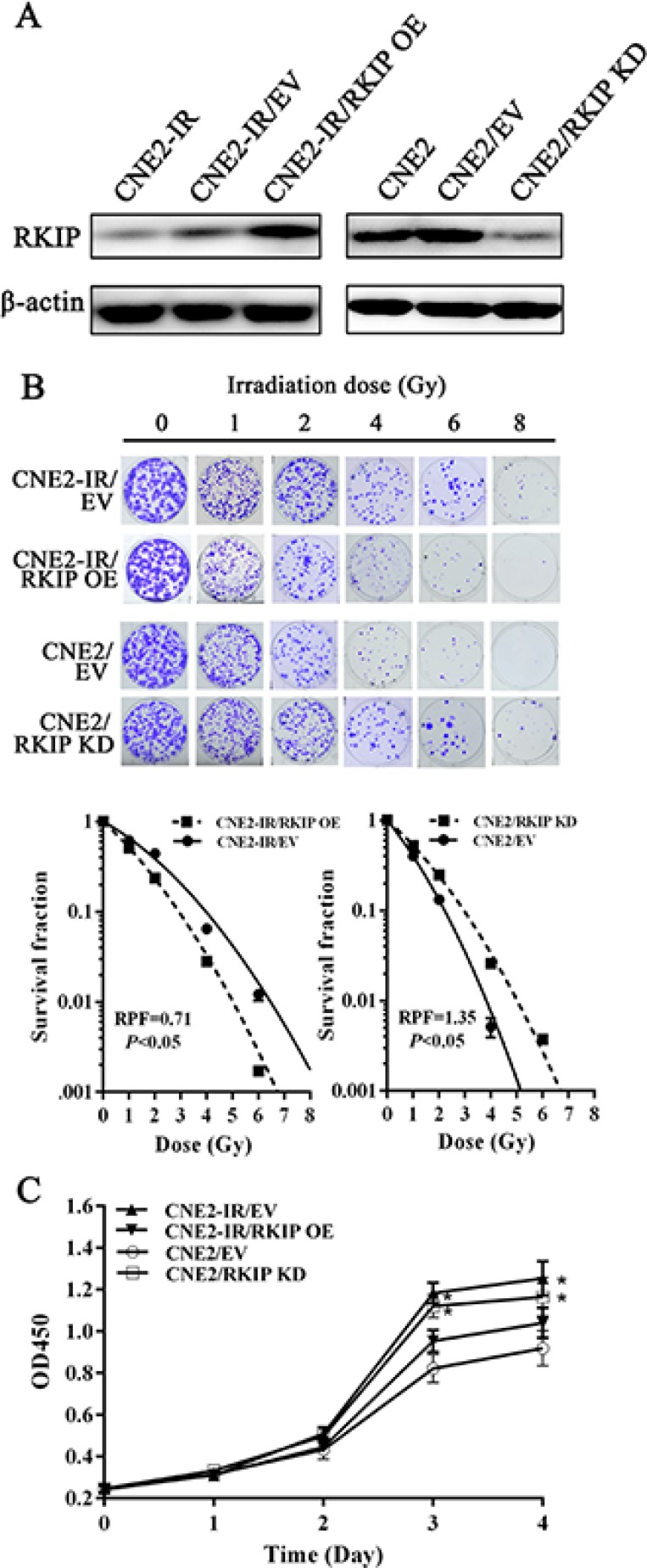
RKIP reduction increases NPC cell radioresistance *in vitro* (**A**) a representative result of Western blotting shows the levels of RKIP expression in the RKIP OE CNE2-IR cells, RKIP KD CNE2 cells and their corresponding EV-transfected cells. (**B**) a clonogenic survival assay shows that radioresponse of RKIP OE CNE2-IR cells, RKIP KD CNE2 cells and their corresponding EV-transfected cells. (top) cells were irradiated with a range of 1–8Gy radiation doses, and colonies that formed after incubation of 12 days were stained with crystal violet and photographed; (bottom) dose survival curves were created by fitting surviving fractions to the linear quadratic equation. (**C**) CCK-8 assay shows that proliferation of RKIP OE CNE2-IR cells, RKIP KD CNE2 cells and their corresponding EV-transfected cells after 4Gy irradiation. Three experiments were done; Means, SDs, and statistical significance are denoted; *P < 0.01. OE, overexpression; KD, knockdown; EV, empty vector.

**Figure 3:**
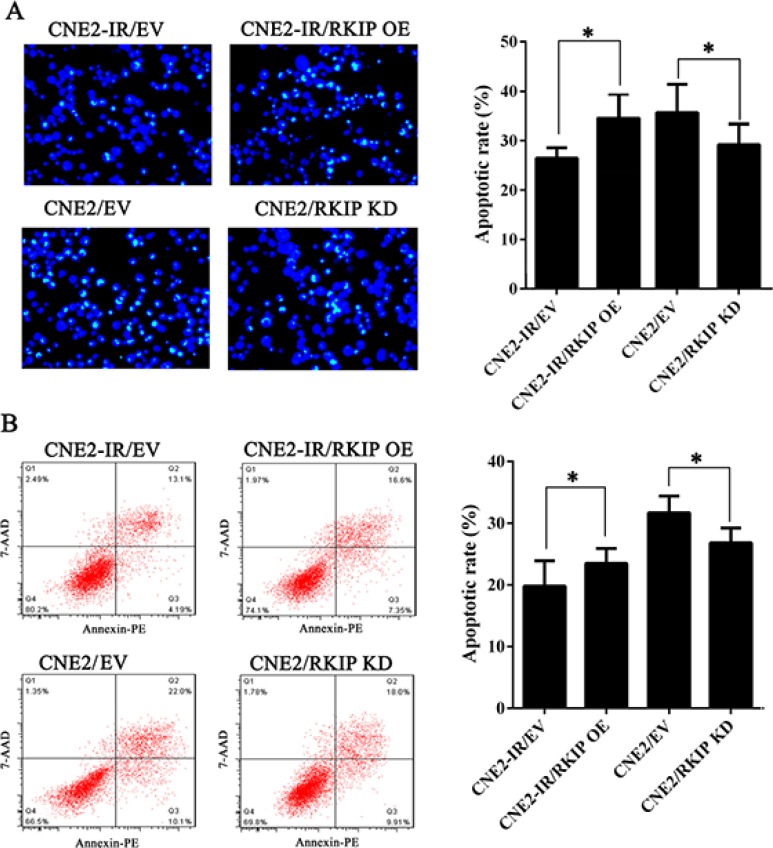
RKIP reduction inhibits irradiation-induced NPC cell apoptosis (**A**) (left) RKIP OE CNE2-IR cells, RKIP KD CNE2 cells and their corresponding EV-transfected cells were irradiated with 6Gy irradiation, incubated for 72 hours, and stained with Hoechst 33258 and photographed; (right) a histogram shows the apoptotic rate of these cells. (**B**) (left) RKIP OE CNE2-IR cells, RKIP KD CNE2 cells and their corresponding EV-transfected cells were irradiated with 6Gy irradiation, incubated for 72 hours, subjected to analysis of apoptosis using flow cytometry, and a representative result of cell apoptosis is showed; (right) a histogram shows the apoptotic rate of these cells. Three experiments were done; Means, SDs, and statistical significance are denoted; *P < 0.01. OE, overexpression; KD, knockdown; EV, empty vector.

To determine the effect of RKIP on NPC cell radioresistance *in vitro*, we established CNE2-IR cell lines with stable RKIP overexpression, CNE2 cell lines with stable RKIP knockdown and their corresponding control cell lines (Figure 2A), and then cell radiosensitivity was determined. A clonogenic survival assay showed that RKIP overexpression decreased CNE2-IR cell radioresistance [AUC 1.33 (RKIP OE) vs. 1.87 (empty vector); *P* < 0.05; RPF = 0.71], whereas RKIP knockdown increased CNE2 cell radioresistance [AUC 1.35 (PKIP KD) vs. 1.00 (empty vector); *P* < 0.05; RPF = 1.35] (Figure 2B). Furthermore, the effect of RKIP on the cell proliferation in response to irradiation was examined by CCK-8 assay. As shown in the Figure 2C, RKIP overexpression inhibited while RKIP knockdown enhanced NPC cell proliferation after 4Gy irradiation. The apoptosis resulting from irradiation is, to a considerable degree, understood as radiosensitivity [31]. Therefore, we analyzed the effect of RKIP on the irradiation-induced apoptosis of NPC cells. Hoechst 33258 staining showed that RKIP overexpression increased irradiation-induced apoptosis of CNE2-IR cells [35.72 ± 5.70% (RKIP OE) vs. 26.46 ± 2.10% (empty vector); *P* < 0.01], whereas RKIP knockdown decreased irradiation-induced apoptosis of CNE2 cells [27.54 ± 3.71% (RKIP KD) vs. 38.47 ± 6.41% (empty vector); *P* < 0.01] (Figure 3A). Flow cytometry analysis also showed that RKIP overexpression increased irradiation-induced apoptosis of CNE2-IR cells [23.57 ± 2.43% (RKIP OE) vs. 19.87 ± 4.15% (empty vector); *P* < 0.01], whereas RKIP knockdown decreased irradiation-induced apoptosis of CNE2 cells [26.82 ± 2.47% (RKIP KD) vs. 31.67 ± 2.65% (empty vector); *P* < 0.01] (Figure 3B). Taken together, these results demonstrate that RKIP reduction promotes NPC cell radioresistance *in vitro*.

### RKIP reduction increases NPC cell radioresistance *in vivo*

**Figure 4:**
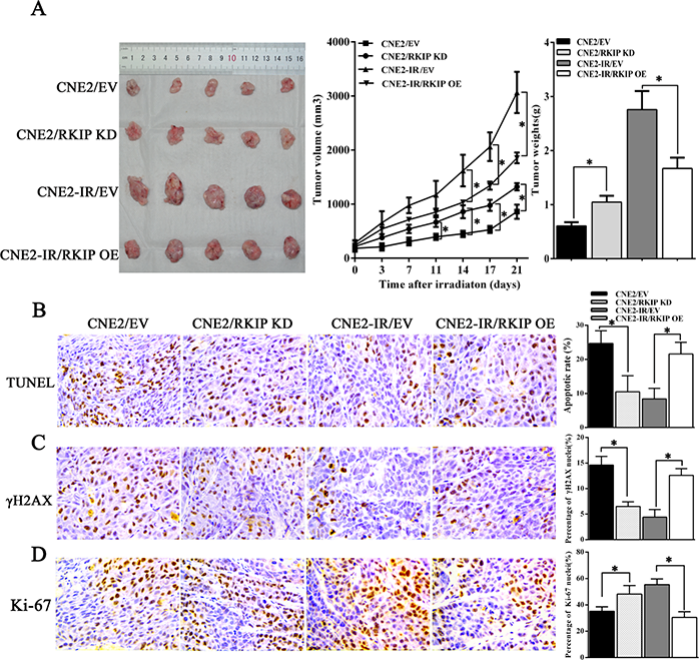
RKIP reduction increases NPC cell radioresistance *in vivo* (**A**) the growth and weight of tumors generated by RKIP KD CNE2 cells, RKIP OE CNE2-IR cells and their corresponding EV-transfected cells after 8Gy irradiation. (left) 3 weeks after irradiation, the mice were killed, and the tumors were photographed; (middle) the growth curves of the tumors after irradiation (*n* = 5 each group) at the sacrifice with respect to the first measurements; (right) the average weights of the tumors after irradiation (*n* = 5 each group) at the sacrifice. (**B**) (left) a representative image of TUNEL detection of apoptotic cells in the tumors generated by RKIP KD CNE2 cells, RKIP OE CNE2-IR cells and their corresponding EV-transfected cells after irradiation; (right) a histogram shows percentages of apoptotic cells in the tumors (*n* = 5 each group). (**C**) (left) a representative image of immunohistochemical staining of γH2AX in the tumors generated by RKIP KD CNE2 cells, RKIP OE CNE2-IR cells and their corresponding EV-transfected cells after irradiation; (right) a histogram shows percentages of γ-H2AX positive cells in the tumors (*n* = 5 each group). (**D**) (left) a representative image of immunohistochemical staining of Ki-67 in the tumors generated by RKIP KD CNE2 cells, RKIP OE CNE2-IR cells and their corresponding EV-transfected cells after irradiation; (right) a histogram shows percentages of Ki-67 positive cells in the tumors (*n* = 5 each group). Means, SDs, and statistical significance are denoted; *P < 0.01. Original magnification, ×400. OE, overexpression; KD, knockdown; EV, empty vector.

To determine the effect of RKIP on NPC radioresistance *in vivo*, we generated subcutaneous tumors in nude mice using NPC cells with RKIP expression changes, and then tumor radioresponse was assessed after receiving total 8Gy irradiation. The results showed that radioresistance of tumors generated by RKIP overexpression CNE2-IR cells was significantly lower than that of tumors generated by control CNE2-IR cells, whereas radioresistance of tumors generated by RKIP knockdown CNE2 cells was significantly higher than that of tumors generated by control CNE2 cells as demonstrated by tumor growth and weight measurement (Figure 4A). TUNEL assay showed that RKIP knockdown decreased while RKIP overexpression increased the number of apoptotic cells in the xenograft tumors [10.55 ± 4.71% (RKIP KD) vs. 24.72 ± 3.70% (empty vector); *P* < 0.01] [21.67 ± 3.41% (RKIP OE) vs. 8.44 ± 3.10% (empty vector); *P* < 0.01] (Figure 4B). Immunohistochemical staining showed that RKIP knockdown decreased while RKIP overexpression increased the number of γH2AX positive cells, *i.e.* cells with DNA damage in the xenograft tumors [6.58 ± 0.92% (RKIP KD) vs. 14.64 ± 1.77% (empty vector); *P* < 0.01] [12.66 ± 1.36% (RKIP OE) vs. 4.47 ± 1.56% (empty vector); *P* < 0.01] (Figure 4C). Moreover, RKIP knockdown increased while RKIP overexpression decreased the number of Ki-67 positive cells, *i.e.* proliferation cells in the xenograft tumors [47.35 ± 7.6% (RKIP KD) vs. 36.43 ± 3.57% (empty vector); *P* < 0.01] [32.30 ± 4.42% (RKIP OE) vs. 54.97 ± 5.27% (empty vector); *P* < 0.01] (Figure 4D). Taken together, these results demonstrate that RKIP reduction promotes NPC cell radioresistance *in vivo*.

### RKIP-regulated NPC cell radioresponse is mediated by ERK and AKT signaling

**Figure 5:**
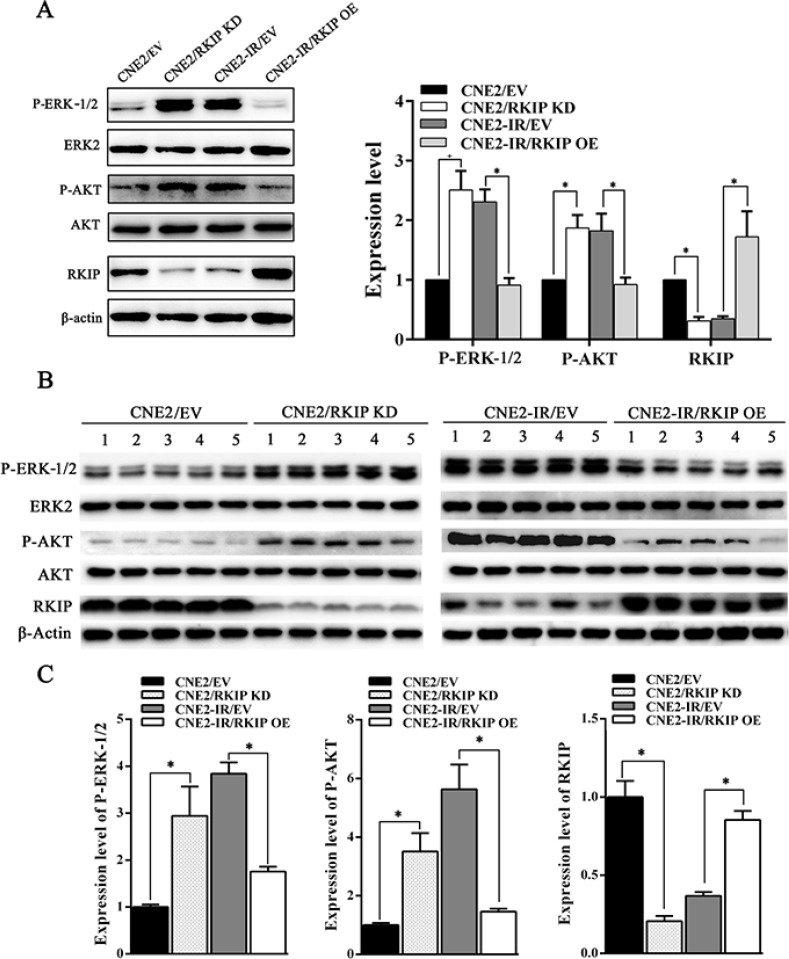
RKIP inhibits activity of ERK-1/2 and AKT signaling in the irradiated NPC cells and xenograft tumors (**A**) (left) a representative result of Western blotting shows the levels of phospho-ERK−1/2, phospho-AKT and RKIP in the RKIP KD CNE2 cells, RKIP OE CNE2-IR cells and their corresponding EV-transfected cells after 4Gy irradiation; (right) a histogram shows the levels of phospho-ERK−1/2, phospho-AKT and RKIP in these cells. (**B**) (top) a representative result of Western blotting shows the levels of phospho-ERK−1/2, phospho-AKT and RKIP in the tumors generated by RKIP KD CNE2 cells, RKIP OE CNE2-IR cells and their corresponding EV-transfected cells after 8Gy irradiation; (bottom) a histogram shows the expression levels of phospho-ERK-1/2, phosphor-AKT and RKIP in the xenograft tumors. Means, SDs, and statistical significance are denoted; *P < 0.01. OE, overexpression; KD, knockdown; EV, empty vector.

**Figure 6:**
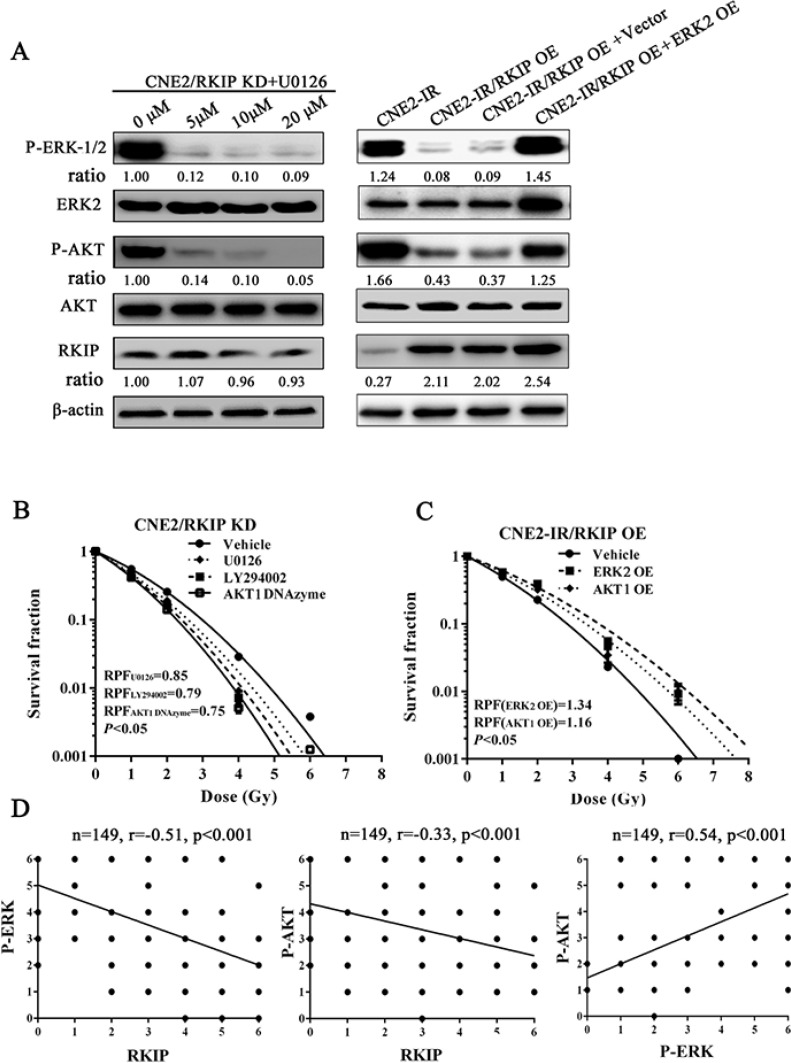
RKIP-regulated NPC cell radioresponse is mediated through ERK and AKT signaling (**A**) (left) a representative result of Western blotting shows the levels of the phospho-ERK-1/2, phospho-AKT and RKIP in the RKIP KD CNE2 treated with a range of 5–20 μmol/L MEK inhibitor U0126; (right) a representative result of Western blotting shows the levels of phospho-ERK-1/2, phospho-AKT and RKIP in the RKIP OE CNE2-IR cells transfected with 4 μg/mL ERK-2 expression vector (OE). (**B**) a clonogenic survival assay shows that inhibition of ERK and AKT signaling significantly abrogated CNE2 KD cell radioresistance induced by RKIP knockdown. Cells treated with 10 μmol/L U0126, 10 μmol/L LY294002 or 2 μmol/L AKT-1 DNAzyme were irradiated with a range of 1–8Gy radiation doses, and dose survival curves were created by fitting surviving fractions to the linear quadratic equation. (**C**) a clonogenic survival assay shows that activation of ERK and AKT signaling restored CNE2-IR OE cell radioresistance reduced by RKIP overexpression. Cells transfected with 4 μmol/L of ERK-2 or AKT-1 expression plasmid were irradiated with a range of 1–8Gy radiation doses, and dose survival curves were created by fitting surviving fractions to the linear quadratic equation. (C) Spearman rank correlation analysis shows that RKIP level was negatively associated with phospho-ERK−1/2 level (a) and phospho-AKT level (b), and phospho-ERK−1/2 level was positively associated with phospho-AKT level in 149 cases of NPCs. OE, overexpression; KD, knockdown.

To explore the signaling mechanism of RKIP in NPC radioresistance, we investigated whether RKIP-regulated NPC cell radioresponse is mediated by ERK and AKT signaling. Western blotting showed that the phosphorylated level of ERK−1/2 and AKT significantly increased in the RKIP knockdown CNE2 cells, whereas significantly decreased in the RKIP overexpression CNE2-IR cells as compared to their corresponding control cells after 4Gy irradiation (Figure 5A). Moreover, the expression levels of phospho-ERK−1/2 and phospho-AKT was reduced in the tumors generated by RKIP overexpression CNE2-IR cells, whereas increased in the tumors generated by RKIP knockdown CNE2 cells relative to tumors generated by their corresponding control cells after 8Gy irradiation (Figure 5B and 5C). The results indicate that RKIP inhibits the activation of ERK-1/2 and AKT signaling in the irradiated NPC cells and tumors. Interestingly, treatment of RKIP knockdown CNE2 cells with MEK inhibitor U0126 (Promega, Madison, WI, USA) decreased phospho-AKT level, whereas transfection of ERK-2 expression plasmid into RKIP overexpression CNE2-IR cells significantly increased phospho-AKT level after 4Gy irradiation, suggesting that AKT is a downstream transducer of ERK signaling in the RKIP-regulating signaling pathways of irradiated NPC cells (Figure 6A).

Next, we determined whether RKIP reduction increases NPC radioresistance via ERK and PI3K signaling. A clone survival assay showed that treatment of RKIP knockdown CNE2 cells with U0126 (Promega) or AKT inhibitor LY294002 (CST, Beverly, MA, USA) significantly abrogated cell radioresistance induced by RKIP knockdown [AUC 1.18 (U0126) vs. 1.40 (vehicle), *P* < 0.05; RPF = 0.85] [1.10 AUC (LY294002) vs. 1.40 (vehicle), *P* < 0.05; RPF = 0.79] (Figure 6B); transfection of AKT1 DNAzyme into RKIP knockdown CNE2 cells also significantly abolished cell radioresistance induced by RKIP knockdown [AUC 1.04 (AKT1 DNAzyme) vs. 1.40 (vehicle), *P* < 0.05; RPF = 0.75] (Figure 6B); transfection of ERK-2 or AKT1 expression plasmid into RKIP overexpression CNE2-IR cells restored cell radioresistance reduced by RKIP overexpression [AUC 1.73 (ERK-2 OE) vs. 1.30 (empty vector), *P* < 0.05; RPF = 1.34] [AUC 1.54 (AKT1 OE) vs. 1.30 (empty vector), *P* < 0.05; RPF = 1.16] (Figure 6C). Collectively, these data demonstrate that RKIP-regulated NPC cell radioresponse is mediated through ERK and AKT signaling, and RKIP reduction promotes NPC cell radioresistance by increasing ERK and AKT activity.

In the cohort of NPC tissues, we observed that the levels of phospho-ERK−1/2 and phospho-AKT were significantly higher in the radioresistant NPCs than those in the radiosensitive NPCs (Figure 1A, Table 1). Correlation analyses revealed that RKIP level was negatively associated with phospho-ERK-1/2 level (*r* = −0.51, *P* < 0.001) and phospho-AKT level (*r* = −0.33, *P* < 0.001), and phospho-ERK−1/2 level was positively associated with phospho-AKT level (*r* = 0.54, *P* < 0.001) (Figure 6D). The results indicate that RKIP reduction enhances the radioresistance of NPC patients probably by activating ERK and AKT signaling.

## DISCUSSION

In this study, we found that RKIP was frequently downregulated in the radioresistant NPC tissues, and its reduction correlated with NPC radioresistance and poor patient survival, outlining a potential biomarker for NPC radiosensitization and patient prognosis. To gain insight into RKIP effect on NPC radioresponse, we performed *in vitro* and *in vivo* radioresponse assays with a combination of loss-of-function and gain-of-function approaches, and demonstrate that reduced RKIP enhances radioresistance in the NPC cells and xenograft tumors.

Next, we investigated the signaling mechanism of RKIP-regulated NPC radioresponse. We observed that RKIP knockdown increased while RKIP overexpression decreased the phosphorylated level of ERK−1/2 and AKT in the NPC cells and xenograft tumors after irradiation, indicating that RKIP decreases the activation of ERK and AKT signaling in the irradiated NPC cells *in vitro* and *in vivo*. Moreover, our result also suggests that AKT is a downstream transducer of ERK signaling in the RKIP-regulating signaling pathways.

To confirm that ERK and AKT signaling mediates RKIP-regulated NPC radioresponse, we used pharmacological and genetic approaches to change the activity or expression of ERK and AKT in the NPC cells, and then detected cell radioresponse. The results showed that MEK inhibitor U0126, PI3K inhibitor LY294002 or AKT1 DNAzyme significantly abrogated cell radioresistance induced by RKIP knockdown in the radiosensitive NPC cells, and transfection of ERK-2 or AKT1 expression plasmid restored cell radioresistance reduced by RKIP overexpression in the radioresistant NPC cells. In the clinical NPC samples, the levels of phospho-ERK−1/2 and phospho-AKT were significantly higher in the radioresistant NPCs than those in the radiosensitive NPCs, and negatively associated with RKIP levels. Taken together, our results demonstrate that RKIP-regulated NPC cell radioresponse is mediated through ERK and AKT signaling, and RKIP reduction promotes NPC cell radioresistance by increasing ERK and AKT activity. Futhermore, we observed that RKIP reduction conferred a clear proliferation advantage and apoptotic resistance to NPC cells in response to irradiation: RKIP knockdown increased while RKIP overexpression decreased cell proliferation; RKIP knockdown increased while RKIP overexpression decreased cell apoptosis. These data suggest that RKIP functions as proliferation inhibitor and anti-apoptotic factor in the irradiated NPC cells, which is consistent with RKIP-inhibiting ERK and AKT activity.

Most of radioresistant NPCs with low RKIP expression along with data from the *in vitro* and *in vivo* radioresponse study indicate that RKIP is needed for ionizing radiation to kill NPC cells, and induction of RKIP expression can be an effective adjuvant for radiotherapy of NPCs with RKIP reduction. In fact, Rituximab has been shown to upregulate RKIP and sensitize non-Hodgkin's lymphoma cells to chemotherapeutic-induced apoptosis [32], and nitric oxide [33] and proteasome inhibitor NPI-0052 [23] have been reported to increase tumor cell sensitivity to chemo-immunotherapeutics via inducing RKIP expression and then inhibiting anti-apoptotic pathways. Furthermore, our *in vitro* and *in vivo* data showed that RKIP dwonregulation increased NPC radioresistance by enhancing ERK and AKT signaling activity, suggesting ERK and AKT as possible targets for personalized therapeutic intervention in NPCs with reduced RKIP.

In summary, we demonstrate that RKIP is critical for radioresponse of NPC cells both *in vitro* and *in vivo*, and its reduction correlates with NPC radioresistance and poor patient prognosis, and RKIP reduction promotes NPC radioresistance via increasing ERK and AKT activity. These findings provide strong evidences for RKIP-ERK-AKT signaling axis as a radiosensitizing target in NPCs with reduced RKIP.

## MATERIALS AND METHODS

### Cell lines

Radioresistant human NPC cell line CNE2-IR and radiosensitive cell line CNE2 cells were previously established by us [34], and cultured with RPMI-1640 medium containing 10% FBS (Invitrogen, Carlsbad, CA, USA). Radioresistant CNE2-IR cells were derived from parental CNE2 cells by treating the cells with four rounds of sublethal ionizing radiation [34]. Radiosensitive CNE2, used as a control, were treated with the same procedure except sham irradiated. Experiments were performed with the CNE2-IR cells within 4 to 10 passages after the termination of irradiation, and their radioresistance was tested by a clonogenic survival assay before use.

### Patients and tissue samples

One hundred and forty-nine NPC patients without distant metastasis (M0 stage) at the time of diagnosis who were treated by radical radiotherapy alone in the First Hospital of Chengzhou City (Chengzhou, China) between Jan 2007 and Dec 2009 were recruited in this study. Using a modified linear accelerator, radiotherapy was administered for treatment of NPC patients at 2Gy per fraction per day, 5 days per week, for a total dose of 70Gy. NPC tissue biopsies were obtained at the time of diagnosis before any therapy, fixed in 4% formalin and embedded in paraffin. 30 cases of formalin-fixed and paraffin-embedded normal nasopharyngeal mucosa tissues from the non-cancer patients in the same period were also collected, and used for controls. On the basis of the 1978 WHO classification [35], all tumors were histopathologically diagnosed as poorly differentiated squamous cell carcinomas (WHO type III). The clinical stage of the patients was classified and reclassified according to the 2008 NPC staging system of China [36].

The radiotherapy response was evaluated clinically for primary lesions based on nasopharyngeal fiberscope and magnetic resonance imaging (MRI) or CT (computed tomography) one month after the initiation of radiotherapy according to the following criteria. Radioresistant NPC patients were defined as ones with persistent disease (incomplete regression of primary tumor and/or neck lymphonodes) at > 3 months or with local recurrent disease at the nasopharynx and/or neck lymphonodes at ≤12 months after completion of radiotherapy. Radiosensitive NPC patients were defined as ones without the local residual lesions (complete regression) at > 3 months and without local recurrent disease at >12 months after completion of radiotherapy. Based on the above criteria, one hundred and forty-nine NPC patients were composed of seventy-four radioresistant and seventy-five radiosensitive ones.

The patients were followed up strictly in outpatient clinics: every 3 month for the first year and then every 6 months for the next 2 years, and finally annually. The patients were followed up for a maximum period of eighty months and a median of fifty-eight months. Overall survival (OS) was defined as the interval from the initiation of primary radiotherapy to the date of cancer-related death or when censured at the latest date if patients were still alive. Disease-free survival (DFS) was defined as the time from the completion of primary radiotherapy to the date of pathological diagnosis or clinical evidence of local failure and/or distant metastasis. The clinicopathological parameters of the patients used in the present study are shown in Supplementary Table S1.

### Establishment of NPC cell lines with overexpression or knockdown of RKIP

Recombinant plasmid pcDNA3.1(+)-Flag-RKIP and lentiviral pGV248-puro-RKIP shRNA were previously described [27]. To generate NPC cell lines with RKIP overexpression, pcDNA3.1(+)-Flag-RKIP and control plasmid pcDNA3.1(+) were transfected into radioresistant CNE2-IR cells using Lipofectamine 2000 (Invitrogen) according to the manufacturer's instruction, respectively. To generate NPC cell lines with RKIP knockdown, lentiviral pGV248-puro-RKIP shRNA and control lentiviral pGV248-puro-control shRNA were used to infect radiosensitive CNE2 cells according to the manufacturer's instruction, respectively. Cells were selected using neomycin or puromycin for 2 weeks, and NPC cell lines with stable overexpression or knockdown of RKIP and control cell lines were obtained.

### Clonogenic survival assay

A clonogenic survival assay was performed as previously described by us [34]. Briefly, cells were exposed to a range of radiation doses (1–8Gy), and 12 days after irradiation surviving colonies were stained with 0.5% crystal violet and counted. The survival fraction was calculated as the numbers of colonies divided by the numbers of cells seeded times plating efficiency. Radiation dose-response curves were created by fitting the data to the linear quadratic equation *S* = *e*^−*α*D−*β*D^2^ using GraphPad Prism 5.0, where *S* is the surviving fraction, *α* and *β* are inactivation constants, and *D* is the dose in Gy. The area under the curves (AUC) that represent the mean inactivation dose (MID) was calculated using GraphPad Prism 5.0. The radiation protection factor (RPF) was calculated by dividing the MID of the test cells by the MID of control cells.

### Cell proliferation assay

Cells were seeded into 96-well plates (1.0 × 10^4^ cells/well). After incubation for 24 hours, the cells were irradiated with 4Gy. Cell viability at various time intervals was assessed by cell-counting kit-8 assay (CCK-8) (Beyotime, Shanghai, China) according to the manufacturer's instruction. The absorbance of each well was monitored by a spectrophotometer at 450 nm (A450). Three independent experiments were done in triplicate.

### Cell apoptosis assay

Flow cytometry analysis and Hoechst 33258 staining of apoptotic cells 72 hours after 6Gy irradiation were performed as previously described by us [37].

### *In vivo* tumor radioresponse assay

Nude male mice that were 4 weeks old were obtained from the Laboratory Animal Center of Central South University (Changsha, China) and were maintained under specific pathogen-free conditions. For *in vivo* tumor radioresponse assay, mice (*n* = 5 each group) were subcutaneously injected with 5 × 10^6^ cells/mouse into the right flanks. When the tumor volumes reached approximately 50 mm^3^, using a modified linear accelerator, a total dose of 8Gy ionizing radiation was delivered to the tumor at 2Gy per fraction per day in the 4 consecutive days. 3 weeks after completing irradiation, the mice were killed by cervical dislocation, and their tumors were excised, weighted, and cut in half, with one half fixed and embedded in paraffin for TUNEL and immunohistochemical staining, and the remaining half flash frozen in liquid nitrogen for Western blotting. Tumor volume (in mm^3^) was measured by caliper measurements performed every 4 days and calculated by using the modified ellipse formula (volume = length × width^2^/2).

### Western blotting

Proteins were exacted from cells and tissues using RIPA buffer. An equal amount of protein in each sample was subjected to SDS-PAGE separation, followed by blotting onto a PVDF membrane (Millipore, Billerica, MA, USA). Blots were blocked with 5% nonfat dry milk or 3% BSA for 2 hours at room temperature and then incubated with anti-RKIP antibody (#13006, CST), phospho-ERK−1/2 (Thr183/Tyr185) (#4671, CST), or phospho-AKT (Ser473) (#4060, CST) antibody overnight at 4C°, followed by incubation with HRP-conjugated secondary antibody for 1 hour at room temperature. The signal was visualized with an enhanced chemiluminescence detection reagent (Pierce, Minneapolis, MN, USA). β-actin was detected simultaneously using monoclonal mouse anti-β-actin antibody (Sigma, St Louis, MO, USA) as a loading control.

### Immunohistochemical staining

Immunohistochemical staining of RKIP, phospho-ERK−1/2, phospho-AKT, γH2AX (phospho-S139), a marker for DNA double-strand breaks [38], and Ki-67, a marker for cell proliferation, were performed on the formalin-fixed and paraffin-embedded tissue sections. Briefly, after antigen retrieval tissue sections were incubated with anti-RKIP, anti-phospho-ERK−1/2 (Thr183/Tyr185), anti-phospho-AKT (Ser473), anti-γH2AX (ab2893, Abcom, Cambridge, MA, USA) or anti-Ki-67 antibody (ab15580, Abcom) overnight at 4°C, and then were incubated with biotinylated secondary antibody followed by avidin-biotin peroxidase complex (DAKO, Glostrup, Denmark). Finally, tissue sections were incubated with 3′, 3′-diaminobenzidine (Sigma) and counterstained with hematoxylin. In negative controls, primary antibodies were omitted.

Immunohistochemical staining was assessed and scored by two independent pathologists. Discrepancies were resolved by consensus. Staining intensity was categorized: absent staining as 0, weak as 1, moderate as 2, and strong as 3. The percentage of stained cells was categorized as no staining = 0, < 30% of stained cells = 1, 30∼60% = 2, and > 60% = 3. The staining score (ranging from 0–6) for each tissue was calculated by adding the area score and the intensity score. A combined staining score of ≤ 3 was considered to be low expression; and a score of > 3 was considered to be high expression. Quantitative evaluation of DNA damaged or proliferation cells was done by examining the sections in ten random microscopic fields and counting the number of γH2AX or Ki-67 positive nuclei among 1000 carcinoma cells under the light microscope. The rate of DNA damaged or proliferation cells was expressed as positive cells per 100 cancer cells.

### *In situ* detection of apoptotic cells

Terminal deoxynucleotidyl transferase(TdT)-mediated dUTP nick end labeling (TUNEL) was performed to detect apoptotic cells of formalin-fixed and paraffin-embedded tissue sections of xenograft tumors after irradiation with *In Situ* Cell Death Detection Kit (Roche, Basel, Switzerland) according to the manufacturer's instruction. Quantitative evaluation of apoptotic cells was done by examining the sections in ten random microscopic fields and counting the number of TUNEL-positive cancer cells among 1000 carcinoma cells under the light microscope. The apoptotic index was expressed as positive cells per 100 cancer cells.

### Transient transfection of AKT1 DNAzyme, AKT1 or ERK-2 expression plasmid into NPC cells

Cells were cultured with RPMI-1640 medium containing 10% FBS one day before transfection. The transfection of 2 μmol/L DNAzyme targeting AKT1 (the sequence: 5′-TGGTCCACAGGCTAGCTACAACGA CCTGCGGCC-3′) [39], 4 μg/mL recombinant plasmid pReceiver-M13-AKT1 (EX-A0022-M13; GeneCopoeia, Rockville, MD, USA) or recombinant plasmid pReceiver-M13-ERK-2 (EX-A0354; GeneCopoeia) into NPC cells was performed using oligofectamine reagent (Life technologies, Carlsbad, CA, USA) or Lipofectamine 2000. 48 hours after transfection, cells were subjected to further analysis.

### Statistical analysis

All experiments were carried out at least 3 times. Data were presented as the mean ± standard deviation (SD). Statistical analysis was conducted using SPSS 20.0 software. For comparisons between two groups, a Student *t* test or chi-square test was used. Survival curves were obtained by using Kaplan–Meier method, and comparisons were made by using log-rank test. Univariate and multivariate survival analyses were conducted on all parameters by using Cox proportional hazards regression model. The Spearman rank correlation coefficient was used to determine the correlation between two parameters. *P* values less than 0.05 were considered to be statistically significant.

### Ethics statement

This study was approved by the ethics committee of the First Hospital of Chengzhou City, China. Written informed consent was obtained from all participants in the study. All animal experiments were undertaken in accordance with the Guide for the Care and Use of Laboratory Animals of Central South University, with the approval of the Scientific Investigation Board of Central South University.

## SUPPLEMENTARY MATERIALS TABLE


