# Supplementary material for: Reduced RKIP enhances nasopharyngeal carcinoma radioresistance by increasing ERK and AKT activity
Source: Oncotarget. 2016 Feb 5;7(10):11463–77. doi: 10.18632/oncotarget.7201 (PMC4905486; doi:10.18632/oncotarget.7201)
Supplement: Supplementary file 1 [file oncotarget-07-11463-s001.pdf]

Reduced RKIP enhances nasopharyngeal carcinoma radioresistance by increasing ERK and AKT activity

Supplementary Materials

Supplementary Table S1: The clinicopathological characteristics of 149 patients with nasopharygeal carcinoma

Parameters	No. of patients	%
**Gender**		
Male	110	73.83
Female	39	26.17
**Age (y)**		
< 47	68	45.64
≥ 47	81	54.36
**Primary tumor(T) stage**		
T1–2	74	49.66
T3–4	75	50.33
**Lymph node(N) metastasis**		
N0	63	42.28
N1–3	86	57.72
**Distant metastasis(M)**		
M0	149	100.00
**Clinical stage**		
I–II	41	27.52
III–IV	108	72.48
**Histological grade**		
Non-keratinising carcinoma	149	100.00
**Radiotherapy response**		
Sensitivity	74	49.66
Resistance	75	50.33
